# Altered *Pseudomonas* Strategies to Inhibit Surface *Aspergillus* Colonies

**DOI:** 10.3389/fcimb.2021.734296

**Published:** 2021-10-22

**Authors:** Gabriele Sass, Hasan Nazik, Paulami Chatterjee, Pallabi Shrestha, Marie-Christine Groleau, Eric Déziel, David A. Stevens

**Affiliations:** ^1^ Infectious Disease Research Laboratory, San Jose, CA, United States; ^2^ Centre Armand-Frappier Santé Biotechnologie, Institute National de la Recherche Scientifique (INRS), Laval, QC, Canada; ^3^ Division of Infectious Diseases and Geographic Medicine, Department of Medicine, Stanford University School of Medicine, Stanford, CA, United States

**Keywords:** *Pseudomonas*, *Aspergillus*, iron, intermicrobial competition, rhamnolipids, quorum sensing

## Abstract

*Pseudomonas aeruginosa* and *Aspergillus fumigatus* infections frequently co-localize in lungs of immunocompromised patients and individuals with cystic fibrosis (CF). The antifungal activity of *P. aeruginosa* has been described for its filtrates. Pyoverdine and pyocyanin are the principal antifungal *P. aeruginosa* molecules active against *A. fumigatus* biofilm metabolism present in iron-limited or iron-replete planktonic *P. aeruginosa* culture filtrates, respectively. Using various *P. aeruginosa* laboratory wild-type strains (PA14, PAO1, PAK), we found antifungal activity against *Aspergillus* colonies on agar. Comparing 36 PA14 and 7 PAO1 mutants, we found that mutants lacking both major siderophores, pyoverdine and pyochelin, display higher antifungal activity on agar than their wild types, while quorum sensing mutants lost antifungal activity. Addition of ferric iron, but not calcium or magnesium, reduced the antifungal effects of *P. aeruginosa* on agar, whereas iron-poor agar enhanced antifungal effects. Antifungal activity on agar was mediated by PQS and HHQ, *via* MvfR. Among the MvfR downstream factors, rhamnolipids and elastase were produced in larger quantities by pyoverdine–pyochelin double mutants and showed antifungal activity on agar. In summary, antifungal factors produced by *P. aeruginosa* on agar differ from those produced by bacteria grown in liquid cultures, are dependent on quorum sensing, and are downregulated by the availability of ferric iron. Rhamnolipids and elastase seem to be major mediators of *Pseudomonas*’ antifungal activity on a solid surface.

## Introduction

*P. aeruginosa* and *A. fumigatus* are frequently co-inhabiting airways of cystic fibrosis (CF) individuals and lungs of immune-compromised persons (Smyth and Hurley, 2010; de Bentzmann and Plésiat, 2011; Walsh and Stevens, 2011; Folkesson et al., 2012; Williams and Davies, 2012; Sabino et al., 2015) and have been associated with deterioration of lung function in CF (Schønheyder et al., 1985; Forsyth et al., 1988; Nicolai et al., 1990; Shoseyov et al., 2006; Amin et al., 2010; de Boer et al., 2011; Coughlan et al., 2012; Fillaux et al., 2012; Speirs et al., 2012; Williams and Davies, 2012; Baxter et al., 2013; Ramsey et al., 2014; Mirković et al., 2016).

*P. aeruginosa* and *A. fumigatus* have developed strategies to compete. We have recently reviewed aspects of the physiology of these two organisms with respect to this competition, with a focus on quorum sensing strategies of *P. aeruginosa* (Chatterjee et al., 2020) and their mechanisms of competition for the essential resource, iron (Matthaiou et al., 2018; Chatterjee et al., 2020). Fe^+3^ is the dominant form of iron in oxygenated environments, but acquisition is difficult because of insolubility of ferric compounds.

Under limiting iron conditions, the most prominent antifungal *P. aeruginosa* molecule is pyoverdine (Sass et al., 2017), a siderophore whose main role is to compete for Fe^+3^ in more stringent iron-limiting conditions. Pyochelin (Cornelis and Dingemans, 2013) is the secondary main siderophore, which has 100-fold less binding affinity for Fe^+3^ compared to pyoverdine; it acquires Fe^+3^ when iron is less limited and also has reactive oxygen species-generating capabilities, which can be useful in intermicrobial competition. These Fe^+3^ uptake mechanisms rely on the Fe^+3^ uptake regulator protein, Fur (Pasqua et al., 2017). Alkyl quinolones, especially the *Pseudomonas* quinolone signal 3,4-dihydroxy-2-heptylquinoline (PQS), contribute to antifungal activity, and PQS can also acquire iron (Nazik et al., 2020).

Fe^+2^ is more abundant in low pH or microaerobic environments, and *Pseudomonas* can also take up Fe^+2^ *via* its Fe^+2^-dedicated Feo system, using phenazines to reduce Fe^+3^ (Cornelis and Dingemans, 2013; Nguyen and Oglesby-Sherrouse, 2015). *Pseudomonas* also has mechanisms to lower environmental pO_2_ when iron is scarce, enhancing the availability of Fe^+2^ (Kim et al., 2003). Fe^+2^ enters the bacterial cell *via* porins; then permeases, such as FeoB, enable entry into the cytosol (Nguyen and Oglesby-Sherrouse, 2015). Reliance on Fe^+2^ would become more important if CF lung pathology progresses, and the lung becomes more hypoxic, decreasing the ratio of Fe^+3^/Fe^+2^ (Nguyen and Oglesby-Sherrouse, 2015).

*A. fumigatus* siderophores counteract pyoverdine action efficiently (Matthaiou et al., 2018; Sass et al., 2019). Under non-limiting iron conditions, *P. aeruginosa* no longer releases pyoverdine but uses pyocyanin (5-N-methyl-1-hydroxyphenazine) as a major antifungal molecule (Sass et al., 2021). Finally, *Pseudomonas* toxins, such as exotoxin A, could participate in antifungal activity (Medina-Rojas et al., 2020). All of the studies cited (Sass et al., 2017; Sass et al., 2019; Nazik et al., 2020; Sass et al., 2021) on the direct intermicrobial competition between these two major pathogens were performed using planktonic filtrates of *P. aeruginosa*, and measuring fungal biofilm metabolism as the target. In the present study, we used cocultures of *P. aeruginosa*, *P. aeruginosa* mutants, and *A. fumigatus* on agar surfaces and measured fungal growth. We explore here the interactions under different circumstances than previously studied and show that the actors appear to be different when studying growths on a solid substrate.

## Materials and Methods

### Materials

Pyoverdine, pyocyanin, PQS, HHQ, FeCl_3_, Fe(II) sulfate heptahydrate, rhamnolipids, elastase, elastin congo red (ECR), cetyltrimethylammonium bromide (CTAB), methylene blue (MB), protease inhibitor cocktail (4-(2-aminoethyl)benzenesulfonyl fluoride hydrochloride, AEBSF at 2 mM, aprotinin at 0.3 μM, bestatin at 116 μM, E-64 at 14 μM, leupeptin at 1 μM, and EDTA at 1 mM), and RPMI 1640 medium were purchased from Sigma-Aldrich (St. Louis, MO). RPMI medium was selected because of its fully defined nature, enabling full manipulation of contents when desired; it has a minute amount of iron, it is well buffered, and its physiologic pH and buffering capacity enables its use, when desired, to coculture mammalian cells with whatever microbes or microbial products one wishes to study. Mono- and di-rhamnolipid preparations were produced and purified as before (Tremblay et al., 2007). Trypticase soy agar (TSA) and 6-mm paper disks were purchased from Becton Dickinson (Sparks, MD, USA). Bacto agar was obtained from Carolina Biological Supply Co., Burlington, NC, USA. For all studies on agar, 40- or 100-mm diameter plastic Petri dishes were used (E&K Scientific, Santa Clara, CA, USA).

### Special Agar Preparations

RPMI agar: 5 g Bacto Agar was suspended in 100 ml distilled water, autoclaved, and mixed with 350 ml prewarmed RPMI-1640 medium. Agar for protease assays: 0.2% skim milk was added to RPMI agar. Iron-depleted agar (IDA): 1 mM ascorbate and 1 mM ferrozine were added to RPMI agar, as previously described (Nazik et al., 2020). CTAB/MB agar for rhamnolipid quantification: 0.05% CTAB and 0.02% MB were added to RPMI agar. ECR agar for detecting elastase activity: 0.2% (w/v) ECR was added to RPMI agar. Twenty ml agar was poured into 10-cm Petri dishes and allowed to solidify before use.

### Isolates

Wild-type isolates used in this study are shown in **Table 1**.

**Table 1:** Wild-type isolates used in this study.

Organism	Isolate	Description	ATCC	Ref
*A. fumigatus*	10AF	Virulent patient isolate	90240	(Denning et al., 1990; Denning and Stevens, 1991)
*A. fumigatus*	Af293		MYA-4609	(Nierman et al., 2005)
*P. aeruginosa*	PA14	Parental strain of mutants (**Supplementary Table 1**)		(O’Toole and Kolter, 1998; Lee et al., 2006)
*P. aeruginosa*	PAO1	Parental strain of mutants (**Supplementary Table 1**)	15692	
*P. aeruginosa*	PAK		25102	

Mutants based on PA14 or PAO1 are described in **Supplementary Table 1**. All PA14 mutants were provided by Prof. Eric Deziel. Mutants in the PAO1 background were kindly provided by Prof. P. R. Secor, Departments of Microbiology and Medicine, University of Washington, Seattle (PAO1 mutants for *lasR/rhlR*, *pqsA*), and by Prof. Paulo Visca, Department of Sciences, Roma Tre University, Rome, Italy (PAO1 mutants for *pvdA*, *pvdS*, *pvdA/fpvR*, *pvdA/pchD*, *pchD*). The use of all microbes in our lab is approved by the CIMR Biological Use Committee (approval no. 001-03Yr.15).

### Two-Colony Method for Interaction Between Bacteria and Fungi on Agar

Sterile paper disks were placed on agar plates and inoculated with 10 µl of *A. fumigatus* suspension [10^7^ conidia/ml RPMI]. Ten microliters of *P. aeruginosa* suspension [10^9^ cell/ml RPMI] was inoculated at a 15-mm distance from the center of the *A. fumigatus* inoculation point directly onto the agar. When rhamnolipids were added, bacterial colony droplets were placed on paper disks. When bacteria were placed on paper disks, e.g., in experiments involving rhamnolipids, their antifungal activity was diminished by about 20%, presumably owing to restriction of diffusion, compared to the antifungal activity observed for the same strains when tested without paper disks. In such experiments, all controls also were placed on paper disks. Plates were incubated at 37°C for 48 h, unless indicated otherwise. To quantify effects of nearby *P. aeruginosa* colony growth on *A. fumigatus* colony growth, a quotient was calculated by dividing the distance from the edge of the filter disk to the end of fungal growth in the direction of the bacterial colony (b in **Supplementary Figure 1A**) by the distance from the edge of the filter disk to the end of fungal growth in the opposite direction (a in **Supplementary Figure 1A**). For undisturbed fungal growth (growth in the presence of 10 µl RPMI) this quotient = 1, for inhibition of fungal growth the quotient would be < 1, and for stimulation of fungal growth the quotient would be > 1.

### Agar on Agar Method for Priming Agars With *P. aeruginosa* Products

RPMI agar (5.5 cm diameter) was cut using a sterile scalpel and put on RPMI agar (10 cm diameter). Ten microliters of *P. aeruginosa* bacterial suspension (10^9^ bacteria/ml) or RPMI was distributed on the top RPMI agar piece. The plates were incubated at 37°C for 48 h. After incubation, plates were centrifuged at 1,000 rpm for 10 min. The top agar piece was removed using a sterile plastic loop and replaced with three 6-mm paper disks. Ten microliters of *A. fumigatus* suspension (10^7^ conidia/ml) was inoculated on each disk. The plates were incubated at 37°C for 48 h, pictures were taken, and the area of growth of *A. fumigatus* was calculated.

### *Pseudomonas* Filtrate Production

*P. aeruginosa* wild-type or mutant bacteria (5 × 10^7^ cells/ml) were incubated in RPMI 1640 medium at 37°C and 100 rpm for 48 h. Bacterial cultures were centrifuged at 500 × *g* for 30 min at room temperature and filtered for sterility (0.22 μm).

### Preparation of *Pseudomonas* Agar Filtrates

Twenty µl *P. aeruginosa* bacteria (10^7^ cells/ml) was inoculated and spread on RPMI agar. Plates were incubated at 37°C for 48 h. RPMI agar with *P. aeruginosa* was transferred to 50-ml centrifuge tubes and centrifuged at 4,000 rpm for 30 min. Supernatants were collected and filter-sterilized (0.22 µm).

### Agar Well Method

Five-millimeter-diameter wells were prepared by using a sterile agar boring tool. Fifty microliters of RPMI, agar filtrate, or planktonic bacterial filtrate was placed in separate wells, and plates were left at room temperature until diffusion of all filtrate into the agar. This procedure was repeated two more times, resulting in 150 µl of RPMI, agar, or planktonic filtrate diffusing into the agar wells. Ten microliters of RPMI agar was then allowed to solidify in each well, and 10 µl of *A. fumigatus* (10^5^ conidia/ml) was inoculated into each well. Plates were incubated at 37°C for 48 h. *A. fumigatus* growth areas were calculated.

### Determination of Fungal Growth

Growth of an *A. fumigatus* colony was determined by its growth area as the function of its radius (r), using the formula r^2^π. Areas of inoculation (wells or disks; 28 mm^2^) were subtracted from the calculated fungal growth area (**Supplementary Figure 1B**).

### Bacterial Mutant Supplementation Experiments

Sterile paper disks were placed on RPMI agar and inoculated with 10 µl of *A. fumigatus* suspension [10^7^ conidia/ml RPMI]. Ten microliters of *P. aeruginosa* suspension [10^9^ cell/ml RPMI] was inoculated at a 15-mm distance from the center of the *A. fumigatus* inoculation point directly onto the agar. Ten microliters of RPMI or test substance dilutions were added to the bacterial suspensions. Plates were incubated at 37°C for 48 h. *A. fumigatus* colony growth was quantified as described under *Two-Colony Method for Interaction Between Bacteria and Fungi on Agar*.

### Testing Pure Molecules Directly Against *A. fumigatus* Growth

Method a): sterile 6-mm paper disks were placed on agar and inoculated with 10 µl of *A. fumigatus* suspension [10^5^ conidia/ml RPMI]. Ten microliters of RPMI or test substance dilutions were added to the disks. Method b): wells with a diameter of 6 mm were punched into the agar. Wells were inoculated with 10 µl of *A. fumigatus* suspension [10^5^ conidia/ml RPMI]. Forty microliters of RPMI or test substance dilutions were added to the wells. Plates were incubated at 37°C for 48 h. *A. fumigatus* colony growth was quantified by measuring the area of fungal growth (**Supplementary Figure 1B**) and subtracting the area of the paper disk or well (28 mm^2^).

### Oil Displacement Assay

This method measures the production of diffusible surface-active molecules (biosurfactants), e.g., rhamnolipids, by bacteria. Ten microliters of bacterial suspensions [10^9^ bacteria/ml RPMI] was placed in the center of 4-cm RPMI agar plates and incubated at 37°C for 24 h. Five hundred microliters of fluid mineral oil (sterile mineral baby oil; CVS, Woonsocket, RI) was distributed to cover the whole plate with the exception of the bacterial colony. Excess oil was discarded. Diameters of oil-free zones were measured after 30 min of incubation at room temperature. The diameters were used to calculate oil displacement zones around the bacterial colonies.

### Quantification of Rhamnolipids by LC/MS

*Pseudomonas* strains were grown on TSA overnight. A suspension was prepared in RPMI at an OD_600_ of 0.4. Ten microliters of this suspension was placed at the center of an RPMI agar plate and incubated for 48 h at 37°C. Cells were then recovered from two plates, suspended in 1 ml of NaCl 0.8%, and serially diluted to perform CFU counts. Agar was scraped from the two plates and put in a tube with 15 ml of HPLC-grade acetonitrile. The tubes were left overnight at 4°C to extract the rhamnolipids. One millimeter was transferred in a microtube and centrifuged at 17,000 x *g* for 10 min. The extract was transferred to an HPLC vial, and rhamnolipids were quantified using liquid chromatography coupled with mass spectrometry (LC/MS) as described previously (Abdel-Mawgoud et al., 2014). Briefly, LC/MS analysis was performed using a Quattro Premier XE Tandem Quadrupole Mass Spectrometer (Waters, Brossard, QC, Canada) equipped with a Z-spray interface using electro-spray ionization in negative mode (ESI-MS/MS). Multiple reaction monitoring (MRM) mode was used to quantify rhamnolipids. The capillary voltage was set at 3.0 kV and the cone voltage at 21 V. The source temperature was kept at 120°C. Nitrogen was used as nebulizing and drying gas at flow rates of 15 and 100 ml/min, respectively. The instrument was interfaced to a Waters 2795 HPLC system equipped with a Kinetex (100 × 4.6 mm) 2.6 µm C8 reversed-phase LC column (Phenomenex, Torrance, CA, USA). The mobile phase was a water (A)/acetonitrile (B) gradient with a constant 4-mM concentration of ammonium acetate and programmed as follows: initial 5% B; 0–1 min, 35% B; 1–2 min, 50% B; 2–5 min, 80% B; 5–6 min, hold 3 min, 100% B; 9–11 min, hold 1 min followed by 3 min of re-equilibration. The HPLC flow rate was 400 µl/min split to 40 µl/min by a tee splitter (Valco Instruments, Houston, TX). In MRM mode, the following transitions were monitored: 649→168 for Rha-Rha-C_10_-C_10_, 503→168 for Rha-C_10_-C_10_ and 271→225 for 16-hydroxyhexadecanoic acid, which was used as an internal standard. The collision energy was set at 16, 24, and 30 V, respectively, and the collision gas flow (argon) was set at 0.35 ml/min.

### Statistical Analysis

Results were analyzed using Student’s *t* test, if two groups were compared, and one-way ANOVA combined with a Tukey’s posttest for multiple comparisons. All data in this study are expressed as a mean ± SD. Each assay was performed with at least three biological and three technical replicates. Representative experiments are shown.

## Results

### Under Coculture Conditions on Agar a *P. aeruginosa* PA14 Mutant Lacking Both Major Siderophores, Pyoverdine, and Pyochelin Is More Inhibitory to *A. fumigatus* Than Its Wild Type

**Figure 1:**
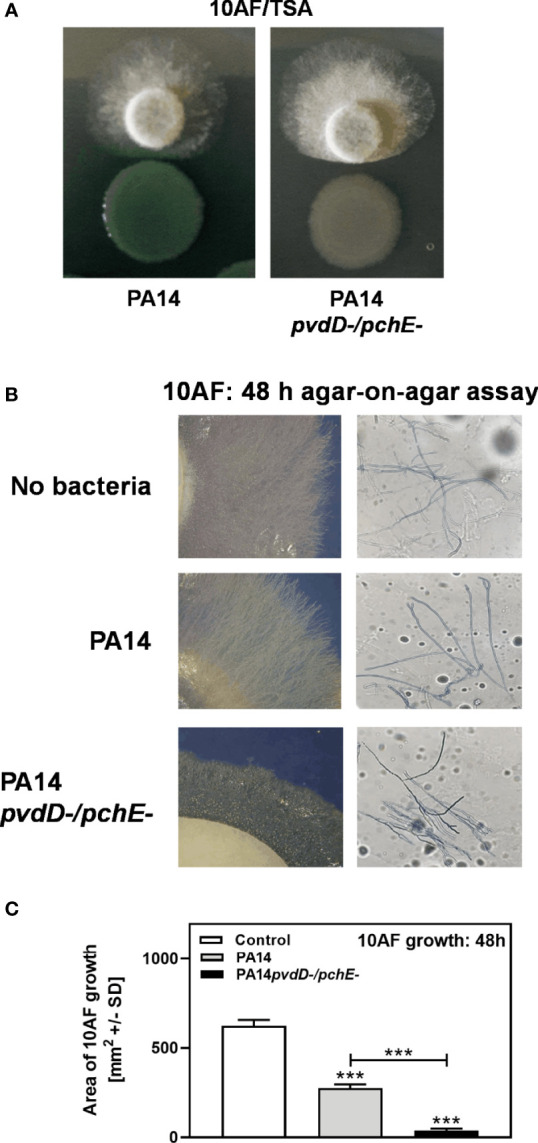
Under coculture conditions on agar, a *P. aeruginosa* PA14 mutant lacking both major siderophores is more virulent for *A. fumigatus* than its wild type. **(A)** Six-millimeter paper disks were placed on RPMI agar. Ten microliters of *A. fumigatus* 10AF conidia (10^7^/ml, prepared in RPMI) was placed on each disk, and 10 µl of PA14 wild type or PA14*pvdD-/pchE-* bacteria (10^9^/ml, prepared in RPMI) was placed 15 mm distant from the disks carrying 10AF. Plates were incubated for 48 h at 37°C, and growth quotients were determined. **(B, C)** A piece of RPMI agar (5.5 cm) was placed on an RPMI agar plate (10 cm). Ten microliters of *P. aeruginosa* bacterial suspension (10^9^ bacteria/ml) or RPMI was distributed on the top RPMI agar piece. Plates were incubated at 37°C for 48 h. After incubation, plates were centrifuged at 1,000 rpm for 10 min. The top agar piece was removed and replaced by three 6-mm paper disks. Ten microliters *A. fumigatus* suspension (10^7^ conidia/ml) was inoculated on each disk. The plates were incubated at 37°C for 48 h, representative pictures were taken **(B)**, and the areas of *A. fumigatus* growth were calculated and are summarized in **(C)** The right part of **(B)** is a magnification of the left part. Agar, primed with PA14*pvdD-/pchE-*, allowed less *A. fumigatus* growth and more hyphal branching, compared to agar primed with PA14 or agar that did not carry bacteria. Comparison in **(C)**: RPMI *vs*. all other bars, or as indicated by the ends of the bracket. Statistical analysis, t-test: Three asterisks = p ≤ 0.001.

We previously reported that, under iron-limiting conditions, planktonic filtrates of *P. aeruginosa* mutants defective in production of the major siderophore pyoverdine inhibit *A. fumigatus* biofilm metabolism to a lesser extent than wild-type filtrates (Sass et al., 2017). Here, we investigated the interaction on a surface (agar). In contrast, a double siderophore-negative pyoverdin-pyochelin mutant of *P. aeruginosa* (PA14*pvdD-/pchE-*) showed stronger antifungal activity than the wild type (**Figure 1A**). Increased effects of coculture with PA14*pvdD-/pchE-* compared to wild type on *A. fumigatus* growth were also visible morphologically. **Figure 1B** compares *A. fumigatus* growth on plates that were primed with the PA14 wild type, or PA14*pvdD-/pchE-* by agar-on-agar incubation. Our results show that agar, primed with PA14*pvdD-/pchE-*, allowed less *A. fumigatus* growth (**Figure 1B**, left side, lowest panel, compared to agar primed with PA14, or without bacterial priming) and more hyphal branching (**Figure 1B**, right side, compared to agar primed with PA14, or without bacterial priming. The right side of **Figure 4B** is a magnification of the left side). Assays, of which one example is shown in **Figure 1B**, were quantified for fungal growth areas (**Figure 1C**).

### Increased Antifungal Effects of PA14pvdD-/pchE Can Be Transferred by Agar-Derived Filtrates, but Not by Planktonic Filtrates

**Figure 2:**
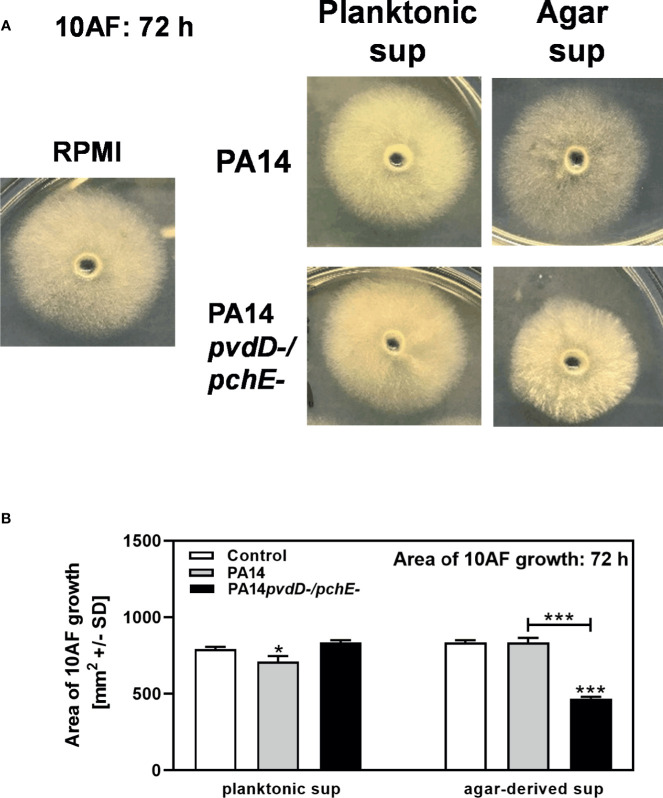
Increased antifungal effects of PA14*pvdD-/pchE-* can be transferred by agar-derived filtrates, but not by planktonic filtrates. **(A, B)** Five-millimeter wells in RPMI agar were inoculated with a total of 150 µl of RPMI, agar, or planktonic bacterial filtrate. Ten microliters of RPMI agar was allowed to solidify in each well, and 10 µl of *A. fumigatus* (10^5^ conidia/ml) was inoculated into each well. Plates were incubated at 37°C for 72 h. The mean diameters of colonies per isolate and treatment were as follows: for RPMI, 32.2 mm; for PA14/planktonic filtrate, 30.7 mm; for PA14*pvdD*-/*pchE*-/planktonic filtrate, 33.2 mm; for PA14/agar filtrate, 33.2 mm; for PA14*pvdD*-/*pchE*-/agar filtrate, 25.2 mm. **(A)** Representative pictures. **(B)** Areas of *A. fumigatus* growth were calculated. Comparison: Control *vs*. all other bars in each group, or as indicated by the ends of the brackets. Statistical analysis, t-test: one, or three asterisks = p ≤ 0.05, and p ≤ 0.001, respectively.

We further prepared sterile filtrates from RPMI agar, or RPMI agar on which PA14 wild type or PA14*pvdD-/pchE-* had grown for 48 h. Comparing effects of these agar filtrates to planktonic PA14 or PA14*pvdD-/pchE-* filtrates, or RPMI on fungal growth after 72 h of incubation, we found that PA14 planktonic filtrates reduced *A. fumigatus* growth, while PA14*pvdD-/pchE-* planktonic filtrates did not much affect *A. fumigatus* growth, compared to the RPMI control (**Figures 2A, B**, left side). *A. fumigatus* colony sizes, incubated with PA14 agar filtrate, did not differ much from colonies incubated with RPMI, or planktonic filtrates, although they appeared less dense (**Figure 2A**). When *A. fumigatus* was incubated with PA14*pvdD-/pchE-* agar filtrate, colony sizes were reduced, whereas colony density seemed increased (**Figure 2A**). **Figure 2B** quantifies results from three experiments of which one is shown in **Figure 2A**. In summary, growth on agar induced production of an antifungal activity by siderophore-deficient *P. aeruginosa*.

### Comparison of Antifungal Activities of *P. aeruginosa* PA14 Mutants Under Coculture Conditions on Agar

**Figure 3:**
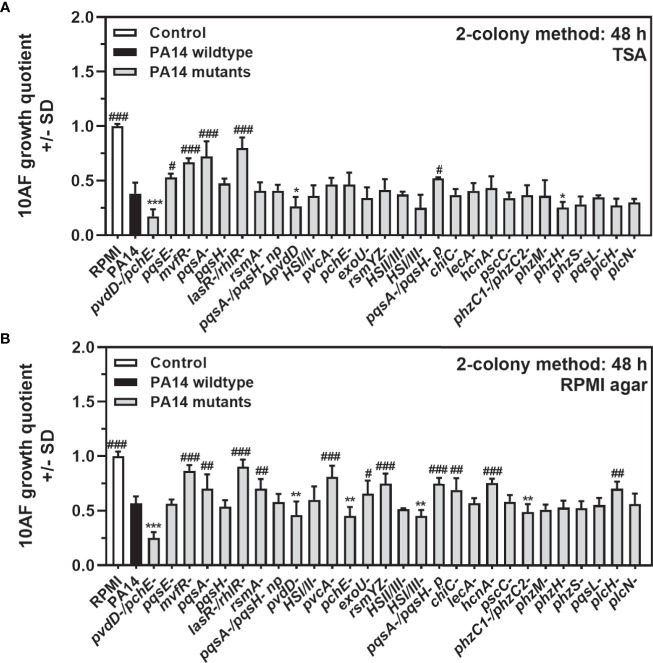
Comparison of antifungal activities of *P. aeruginosa* PA14 mutants under coculture conditions on agar. **(A, B)** Six-millimeter paper disks were placed on RPMI agar. Ten microliters of *A. fumigatus* 10AF conidia (10^7^/ml, prepared in RPMI) was placed on each disk, and 10 µl of PA14 wild-type or 28 mutant bacteria (10^9^/ml, prepared in RPMI) was placed 15 mm distant from the disks carrying 10AF. Plates were incubated for 48 h at 37°C, and growth quotients were determined. Experiments were performed on TSA **(A)** and on RPMI agar **(B)**. Comparison: PA14 (black bar) *vs*. all other bars. Statistical analysis, t-test: one, two, or three asterisks or pound signs = p ≤ 0.05, p ≤ 0.01, and p ≤ 0.001, respectively. Asterisks represent significant increases in antifungal activity; pound signs represent significant decreases.

We next determined growth quotients of the *P. aeruginosa* wild-type reference strain PA14, and 28 PA14 mutants, lacking expression of several functions, including factors important in iron uptake, virulence, or quorum sensing (**Supplementary Table 1**) using the two-colony method, as described in *Materials and Methods*. We observed that in coculture the antifungal activity of most *P. aeruginosa* mutants was either comparable to the wild type, or weaker, with quorum sensing mutants being less antifungal than the wild type, on different agars (**Figure 3A**, assays performed on TSA, and **Figure 3B**, assays performed on RPMI agar). PA14*pvdD-/ΔpchE-*, lacking production of both major siderophores pyoverdine and pyochelin, had a significantly stronger effect than the wild type on *A. fumigatus* growth in cocultures on agar (**Figures 3A, B** **)**. A mutant lacking only pyoverdine (PA14*pvdD-*) had a lesser antifungal effect than the double siderophore mutant, but still was more antifungal than the wild type (**Figures 3A, B** **)**. A mutant only lacking pyochelin (PA14*pchE-*) differed from the wild type in its antifungal activity only on RPMI agar (**Figure 3B**).

**Figure 4:**
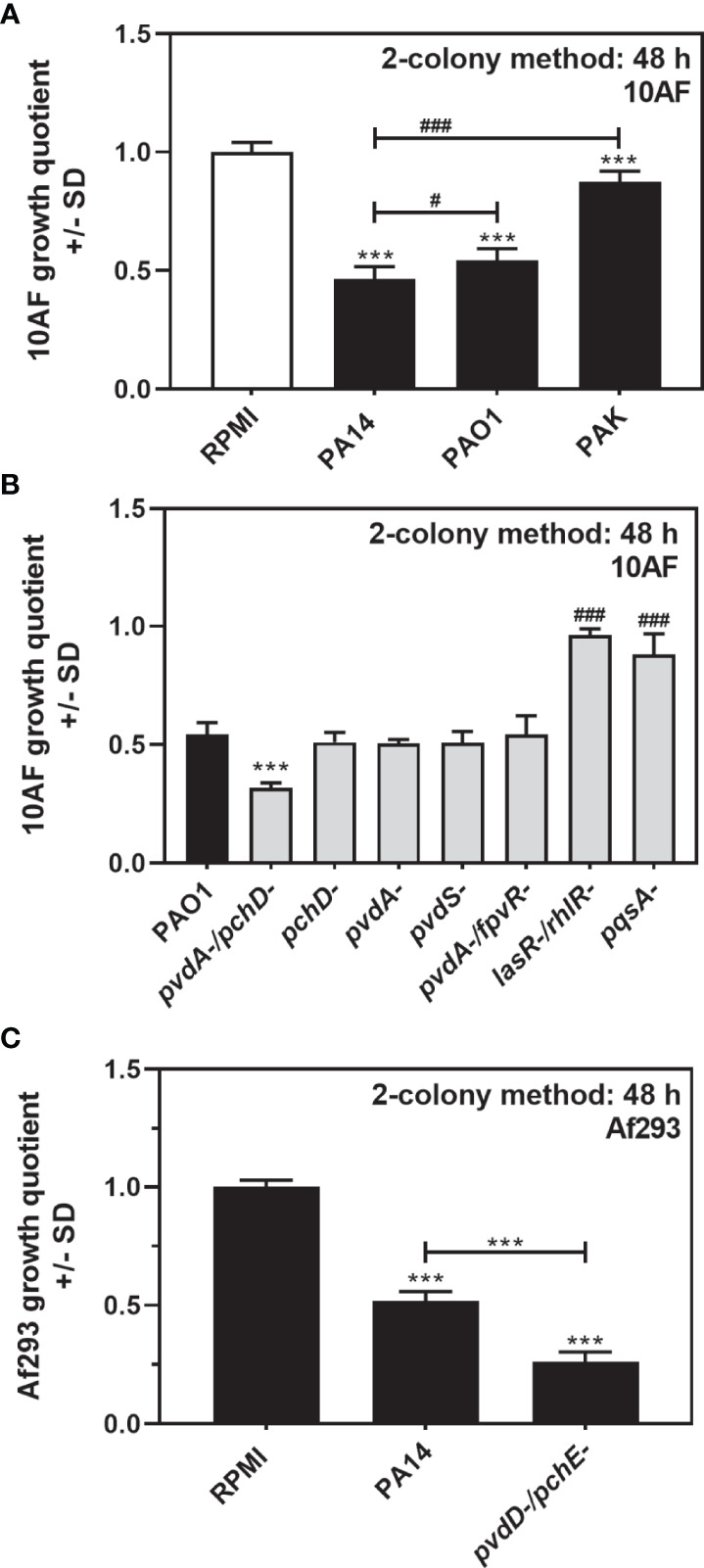
*P. aeruginosa* antifungal activity on agar is not strain-dependent. **(A)** Six-millimeter paper disks were placed on RPMI agar. Ten microliters of *A. fumigatus* 10AF conidia (10^7^/ml, prepared in RPMI) was placed on each disk, and 10 µl of PA14, PAO1, or PAK wild-type bacteria (10^9^/ml, prepared in RPMI) was placed 15 mm distant from the disks carrying 10AF. Plates were incubated for 48 h at 37°C, and growth quotients were determined by the two-colony method. Comparison: RPMI control (white bar) vs. all other bars, or as indicated by the ends of the brackets. **(B)** Six-millimeter paper disks were placed on RPMI agar. Ten microliters of *A. fumigatus* 10AF conidia (10^7^/ml, prepared in RPMI) was placed on each disk, and 10 µl of PAO1 or seven PAO1 mutant bacteria (10^9^/ml, prepared in RPMI) was placed 15 mm distant from the disks carrying 10AF. Plates were incubated for 48 h at 37°C, and growth quotients were determined by the two-colony method. Comparison: PAO1 (black bar) vs. all other bars. **(C)** Six-millimeter paper disks were placed on RPMI agar. Ten microliters of *A. fumigatus* Af293 conidia (10^7^/ml, prepared in RPMI) was placed on each disk, and 10 µl of PA14 wild-type or PA14*pvdD-/pchE-* bacteria (10^9^/ml, prepared in RPMI) was placed 15 mm distant from the disks carrying Af293. Plates were incubated for 48 h at 37°C, and growth quotients were determined by the two-colony method. Comparison: RPMI control *vs*. all other bars, or as indicated by the ends of the bracket. Statistical analysis in **(A–C)**, t-test: one, or three asterisks or pound signs = p ≤ 0.05 and p ≤ 0.001, respectively. Asterisks represent significant increases in antifungal activity; pound signs represent significant decreases.

Antifungal activity on agar, as observed in **Figures 1**, **2** for PA14, was also present when using the *P. aeruginosa* strains PAO1 and PAK (**Figure 4A**). A double siderophore mutant in the PAO1 background (PAO1*pvdA-/pchD-*) was more antifungal on agar than its wild type, whereas the respective single mutants were not (**Figure 4B**). In the PAO1 background, no pyoverdine mutant tested here (*pvdA-*, *pvdS-*, *pvdA-/fpvR-*) was more antifungal than the wild type, whereas both QS mutants (*lasR-/rhlR- and pqsA-*) were less antifungal (**Figure 4B**). Antifungal activity of PA14 on agar and increased antifungal activity of its double siderophore mutant PA14*pvdD-/pchE-* were also present when *A. fumigatus* strains other than 10AF were studied as the target, as shown here for Af293 (**Figure 4C**).

### QS Signaling Contributes to *P. aeruginosa* Antifungal Activity on Agar

**Figure 5:**
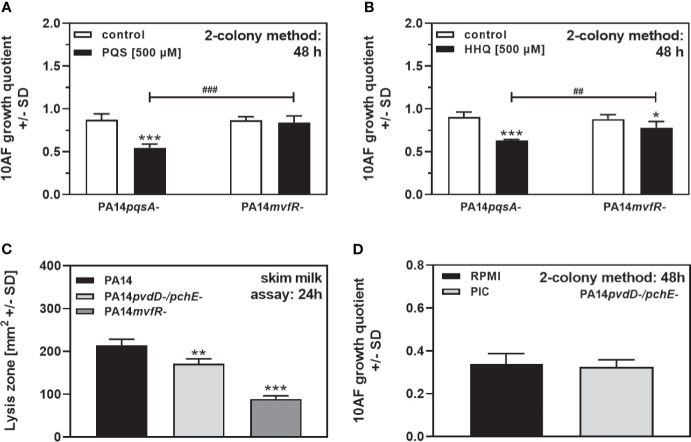
QS-signaling contributes to *P. aeruginosa* antifungal activity on agar. **(A, B)** Six-millimeter paper disks were placed on RPMI agar. Ten microliters of *A. fumigatus* 10AF conidia (10^7^/ml, prepared in RPMI) was placed on each paper disk, and 10 µl of PA14*pqsA- or* PA14*mvfR-* bacteria (10^9^/ml, prepared in RPMI) was placed 15 mm distant from the disks carrying 10AF. Mutant bacterial suspensions were supplemented with RPMI (white bars), PQS (black bars in **A**), or HHQ **(**black bars in **B**). Plates were incubated for 48 h at 37°C, and growth quotients were determined by the two-colony method. Comparison: RPMI controls *vs*. all other bars in the same group, or as indicated by the ends of the brackets. **(C)** Six-millimeter paper disks were placed on RPMI agar, containing 2% skim milk. Ten microliters of PA14, PA14*pvdD-/pchE-*, or PA14*mvfR-* bacteria (10^9^/ml, prepared in RPMI) was placed on the disks. Plates were incubated for 24 h at 37°C, and lysis zones were determined. Comparison: PA14 wild type *vs*. all mutants, or as indicated by the ends of the brackets. **(D)** Six-millimeter paper disks were placed on RPMI agar. Ten microliters of *A. fumigatus* 10AF conidia (10^7^/ml, prepared in RPMI) was placed on each paper disk, and 10 µl of PA14*pvdD-/pchE-* bacteria (10^9^/ml, prepared in RPMI) was placed 15 mm distant from the disks carrying 10AF. Ten microliters of protease inhibitor cocktail (PIC; 1:1000) was added to the bacterial droplets. Plates were incubated at 37°C for 48 h, and growth quotients were determined by the two-colony method. Statistical analysis in **(A–D)**, t-test: one, two, or three asterisks or pound signs = p ≤ 0.05, p ≤ 0.01, and p ≤ 0.001, respectively. Asterisks represent significant increases; pound signs represent significant decreases in antifungal activity **(A**, **B)**, or in proteolytic activity **(C)**.

**Figure 4** revealed that QS mutants (e.g., *pqs* mutants) were severely impaired in their antifungal activity on agar. Mutations blocking pathways leading to PQS and HHQ production (PA14 mutants in *pqsA* or *mvfR*) or mutants in other QS regulators (*lasR-/*Δ*rhlR-*) showed significantly reduced antifungal activity on agar (**Figure 3**). In order to investigate the contribution of PQS and HHQ signaling to antifungal activity on agar, we supplemented PA14*pqsA* or PA14*mvfR* with PQS or HHQ and measured antifungal activity. Our results show that both PQS and HHQ overcame the loss of antifungal activity for the PA14*pqsA* mutant, but not for the PA14*mvfR* mutant (compare **Figures 5A, B** **)**. These data indicate that a PQS and HHQ-dependent “downstream” factor of *mvfR* is important for antifungal activity on agar.

Molecules downstream of *mvfR* could be, e.g., phenazines, proteases, or rhamnolipids (Déziel et al., 2005; Diggle et al., 2006). PA14 as well as its double siderophore mutant produced large amounts of proteases, whereas the PA14Δ*mvfR* mutant produced significantly less proteases under our conditions (**Figure 5C**). We did not find increased protease production by PA14*pvdD-/pchE-*, compared to PA14, indicating that proteases detected in the skim milk assay are not responsible for the increased antifungal activity of the double siderophore mutant (**Figure 5C**). We further added a broad-spectrum protease inhibitor cocktail to growing colonies of PA14*pvdD-/pchE-*, but did not see a loss in antifungal activity (**Figure 5D**).

### Rhamnolipids Have Antifungal Activity on Agar

**Figure 6:**
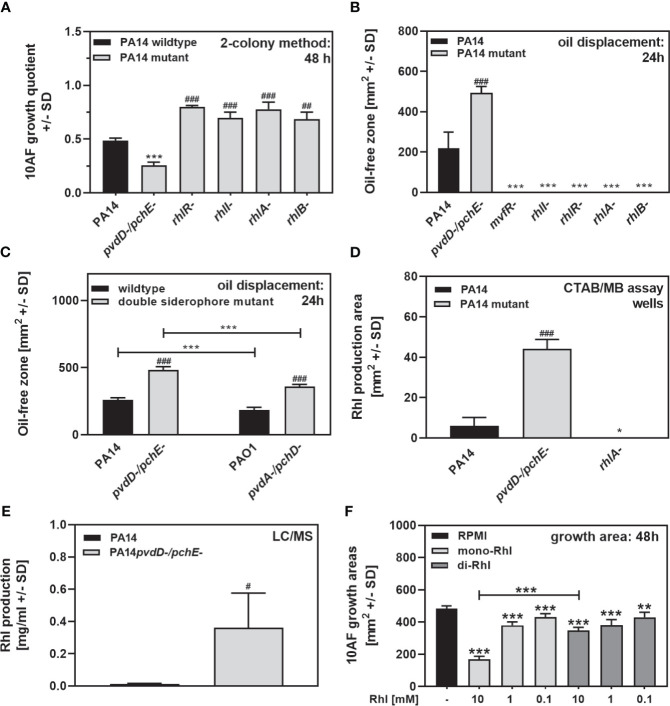
Rhamnolipids have antifungal activity on agar. **(A)** Six-millimeter paper disks were placed on RPMI agar. Ten microliters of *A. fumigatus* 10AF conidia (10^7^/ml, prepared in RPMI) was placed on each disk, and 10 microliters of PA14 wild-type, PA14*pvdD-/pchE-*, PA14*rhlR-*, PA14*rhlI-*, PA14*rhlA*-, or PA14*rhlB-* bacteria (10^9^/ml, prepared in RPMI) was placed 15 mm distant from the disks carrying 10AF. Plates were incubated for 48 h at 37°C, and growth quotients were determined by the two-colony method. Statistical analysis: Comparison: PA14 (black bar) vs. all other bars. **(B)** Ten-microliter droplets of PA14 wild-type, PA14*pvdD-/pchE*-, PA14*mvfR-*, PA14*rhlI-*, PA14*rhlR-*, PA14*rhlA-*, or PA14*rhlB-* bacteria (10^9^/ml, prepared in RPMI) were placed in the middle of 4-cm RPMI agar plates. Plates were incubated at 37°C for 24 h. An oil displacement test was performed. Comparison: PA14 (black bar) *vs*. all other bars. **(C)** Ten-microliter droplets of PA14 wild-type, PA14*pvdD-/pchE-*, PAO1 wild-type, or PAO1*pvdA-/pchD-* bacteria (10^9^/ml, prepared in RPMI) were placed in the middle of 4-cm RPMI agar plates. Plates were incubated at 37°C for 24 h. An oil displacement test was performed. Comparison: PA14 (black bar) *vs*. all other bars, or as indicated by the ends of the brackets. **(D)** Six-millimeter paper disks were placed on CTAB/MB agar. Ten microliters of PA14 wild-type, PA14*pvdD-/pchE-*, or PA14*rhlA-* bacteria (10^9^/ml, prepared in RPMI) was placed on the disks. Plates were incubated for 72 h at 37°C, and blue zones arround the disks were determined. Statistical analysis: t-test: Comparison: PA14 (black bar) *vs*. all other bars. **(E)** Rhamnolipid production by PA14 wild-type and PA14*pvdD-/pchE-* was quantified by LC/MS as described in detail in *Materials and Methods*. **(F)** Six-millimeter paper disks were placed on RPMI agar. Ten microliters of *A. fumigatus* 10AF conidia (10^7^/ml, prepared in RPMI) was placed on each disk, and 10 µl of pure mono-ordi-rhamnolipids was added to the filter disks at 0.1, 1, or 10 mM. Plates were incubated at 37°C for 48 h, and fungal growth areas were determined. Comparison: PA14 (black bar) *vs*. all other bars, or as indicated by the ends of the bracket. Statistical analysis in **(A–F)**, t-test: one, two, or three asterisks or pound signs = p ≤ 0.05, p ≤ 0.01, and p ≤ 0.001, respectively. Asterisks represent significant increases; pound signs represent significant decreases in antifungal activity **(A, F)** or in rhamnolipid production **(B–E)**.

Rhamnolipids under our culture conditions are downstream products of *mvfR*, and rhamnolipid production is deficient in PA14*lasR-/rhlR-*, which showed a loss of antifungal activity on agar (**Figures 3A, B** **)**. PA14 mutants defective in other genes leading to rhamnolipid production also lost substantial antifungal activity on agar (**Figure 6A**, PA14 mutants for *rhlR*, *rhlI*, *rhlA*, and *rhlB*). PA14 mutants defective in *mvfR*, *rhlI*, *rhlR*, *rhlA*, or *rhlB* appeared to produce much less rhamnolipids on RPMI agar (**Figure 6B**). PA14 or PAO1 mutants, defective in major siderophore production, produced more rhamnolipids than their wild types, as shown by oil displacement assays (**Figures 6B, C** **)**, methylene blue agar assays (**Figure 6D**), and LC/MS analysis (**Figure 6E**). As a side note, PAO1 produced less rhamnolipids than PA14 (**Figure 6C**), which corresponds to inferior antifungal activity of PAO1 on agar (**Figure 4A**). Pure rhamnolipids induced dose-dependent antifungal activity on agar, with pure mono-rhamnolipids having stronger effects (**Figure 6F**).

### Elastase Has Antifungal Activity on Agar

**Figure 7:**
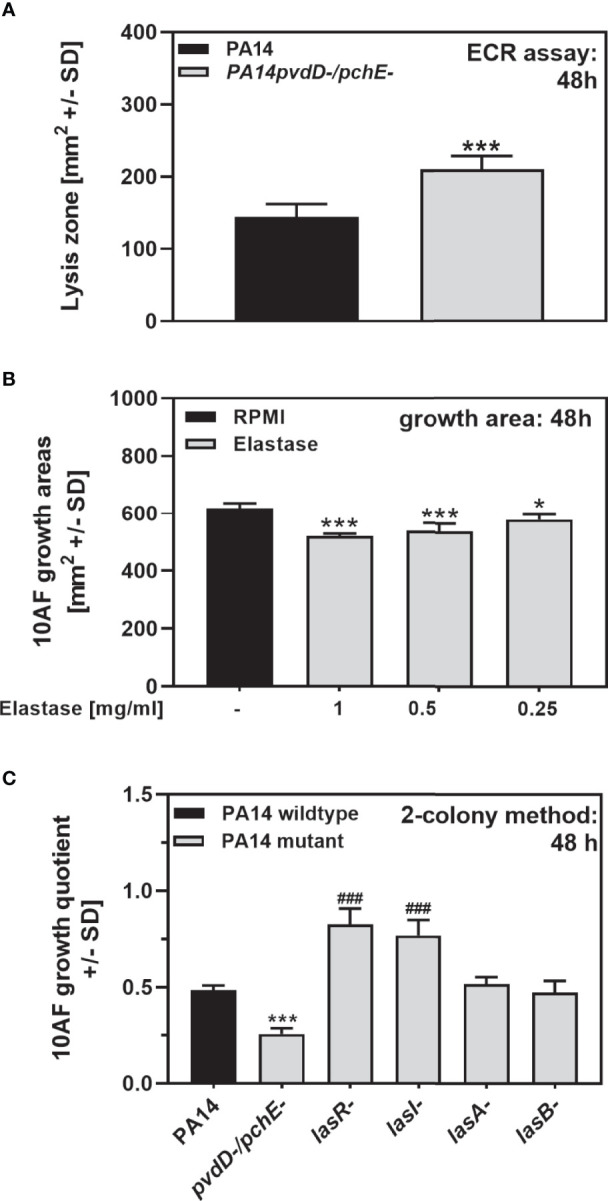
Elastase has antifungal activity on agar. **(A)** Six-millimeter paper disks were placed on ECR agar. Ten microliters of PA14 wild-type or PA14*pvdD-/pchE-* bacteria (10^9^/ml, prepared in RPMI) was placed on the disks. Plates were incubated for 48 h at 37°C, and elastin lysis zones around the disks were determined. **(B)** Six-millimeter paper disks were placed on RPMI agar. Ten microliters of *A. fumigatus* 10AF conidia (10^7^/ml, prepared in RPMI) was placed on each disk, and 10 µl of pure elastase was added to the filter disks at 1, 0.5, or 0.25 mg/ml. Plates were incubated at 37°C for 48 h, and fungal growth areas were determined. Comparison: PA14 (black bar) *vs*. all other bars. **(C)** Six-millimeter paper disks were placed on RPMI agar. Ten miroliters of *A. fumigatus* 10AF conidia (10^7^/ml, prepared in RPMI) was placed on each disk, and 10 µl of PA14 wild-type, PA14*pvdD-/pchE-*, PA14*lasR-*, PA14*lasI-*, PA14*lasA-*, or PA14*lasB-* bacteria (10^9^/ml, prepared in RPMI) was placed 15 mm distant from the disks carrying 10AF. Plates were incubated for 48 h at 37°C, and growth quotients were determined. Comparison: PA14 (black bar) *vs*. all other bars. Statistical analysis in **(A–C)**, t-test: one or three asterisks or pound signs = p ≤ 0.05 and p ≤ 0.001, respectively. Asterisks represent significant increases; pound signs represent significant decreases in elastase production **(A)** or in antifungal activity **(B, C)**.

The PA14 *lasR/rhlR* quorum-sensing double mutant, deficient in production of both elastase (LasB) and rhamnolipids, was significantly inferior in antifungal activity, compared to the wild type (**Figures 3A, B** **)**, indicating that either molecules could contribute to antifungal activity on agar. In fact, we found that PA14*pvdD-/pchE-*, being more antifungal than the wild type, produced more elastolytic activity (**Figure 7A**). Pure elastase inhibited *A. fumigatus* growth at concentrations of ≥0.25 mg/ml (**Figure 7B**). On the other hand, although a loss of *lasR* and *lasI* significantly interfered with PA14 antifungal activity, mutations in *lasA* (coding for a protease) and *lasB* did not (**Figure 7C**), indicating that a LasR-regulated pathway, but not necessarily elastase itself, might be contributing to antifungal activity on agar.

### Rhamnolipids and Elastase Complement the Loss of Antifungal Activity by Their Respective Mutants, and in Combination Increase Antifungal Activity of PA14 Wild Type

**Figure 8:**
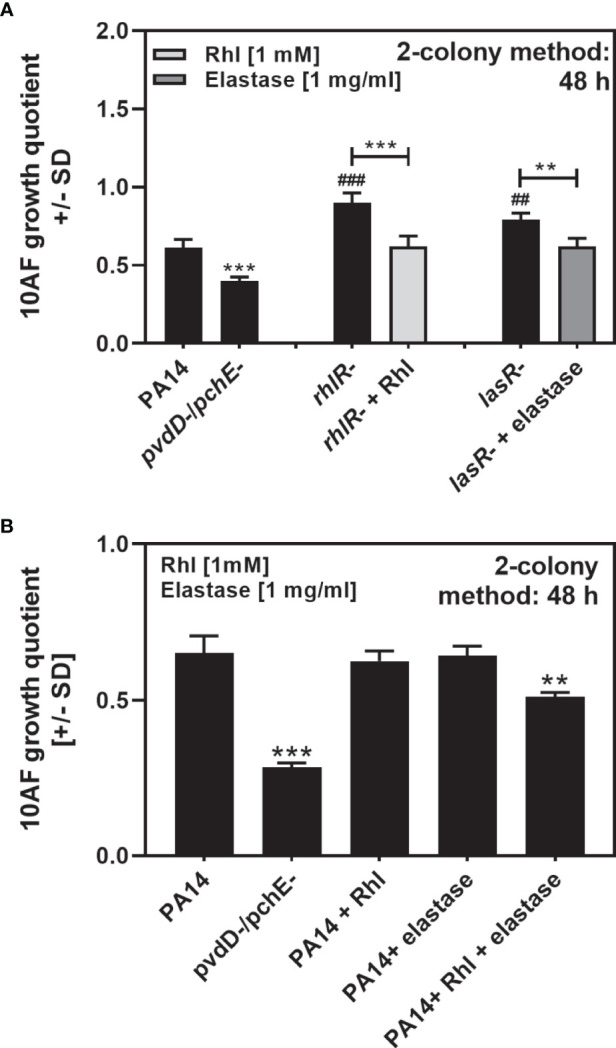
Rhamnolipids and elastase complement the loss of antifungal activity by their respective mutants, and in combination increase antifungal activity of PA14 wild type. **(A, B)** Six-millimeter paper disks were placed on RPMI agar. Ten microliters of *A. fumigatus* 10AF conidia (10^7^/ml, prepared in RPMI) was added to these paper disks. **(A)** Ten microliters of PA14, PA14*pvdD-/pchE-*, PA14*lasR-*, *or* PA14*rhlR-* bacteria (10^9^/ml, prepared in RPMI) was placed on paper disks 15 mm distant from the disks carrying 10AF. Bacterial suspensions were supplemented with 10 µl RPMI (for PA14 or PA14*pvdD-/pchE-*), 10 µl elastase (of 1 mg/ml for PA14*lasR-*), or 10 µl rhamnolipids (of 1 mM, for PA14*rhlR-*). **(B)** Ten microliters of PA14 or PA14*pvdD-/pchE-*bacteria (10^9^/ml, prepared in RPMI) was placed on paper disks 15 mm distant from the disks carrying 10AF. Bacterial suspensions were supplemented with 10 µl RPMI (for PA14 or PA14*pvdD-/pchE-*), 5 µl RPMI plus 5 μl elastase (of 2 mg/ml), 5 µl RPMI plus 5 µl rhamnnolipids (of 2 mM), or 5 ul elastase plus 5 µl rhamnolipids, keeping the amounts of elastase and rhamnolipids identical to **(A)** Plates were incubated at 37°C for 48 h, and growth quotients were determined by the two-colony method. Comparison: RPMI controls *vs*. all other bars, or as indicated by the ends of the brackets. Statistical analysis, t-test: two, or three asterisks or pound signs = p ≤ 0.01 and p ≤ 0.001, respectively. Asterisks represent significant increases; pound signs represent significant decreases in antifungal activity.

Mutants for LasR or RhlR showed diminished antifungal activity on agar (**Figures 6A**, **7C**). Addition of rhamnolipids or elastase to their respective mutants during growth restored antifungal activity to that seen for the PA14 wild type, but did not further increase antifungal activity to levels seen for the PA14 double siderophore mutant (**Figure 8A**). These data support the contribution of rhamnolipids and elastase to antifungal activity on agar. We observed that the combined loss of siderophores pyoverdine and pyochelin, resulting in iron deficiency to this mutant, increased rhamnolipid and elastase production (**Figures 6C–E**, **7A**), and increased antifungal activity of that mutant on agar over wild type. When adding rhamnolipids and elastase to the PA14 wild type, we found that combined addition, but not addition of just one agent, increased antifungal activity over the wild type (**Figure 8B**).

### Iron Modulates Antifungal Activity of *P. aeruginosa on* Agar

**Figure 9:**
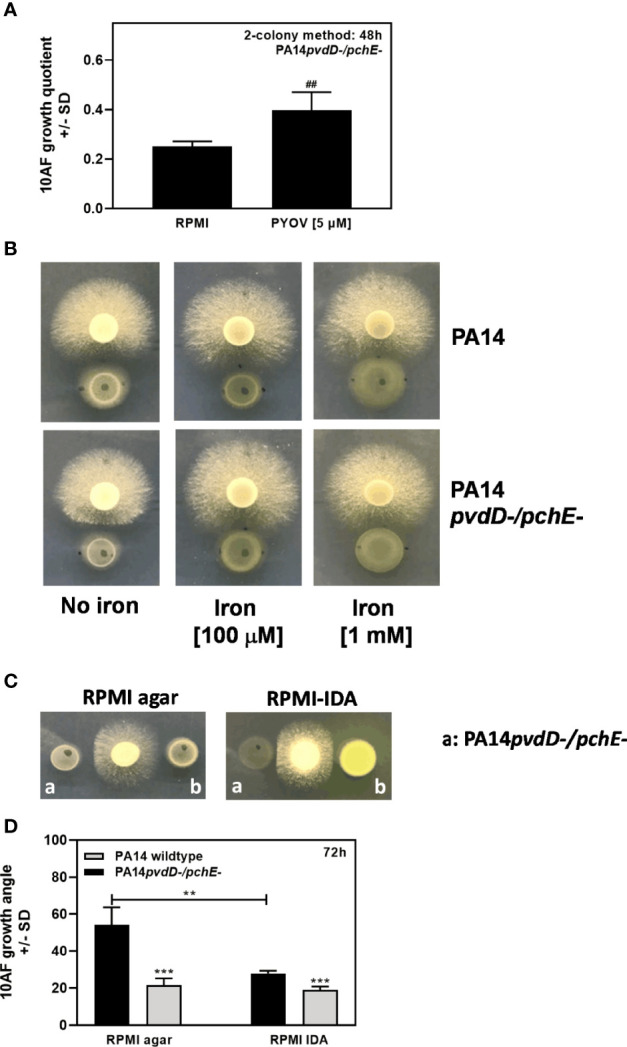
Effects of pyoverdine or iron modification on antifungal activity on agar. **(A)** Six-millimeter paper disks were placed on RPMI agar. Ten microliters of *A. fumigatus* 10AF conidia (10^7^/ml, prepared in RPMI) was placed on each disk, and 10 µl of *P. aeruginosa* bacteria (10^9^/ml, prepared in RPMI) was placed 15 mm distant from the disks carrying 10AF. RPMI or 10 µl of 5 µM pyoverdine in RPMI was added to the bacterial suspension drops. Plates were incubated for 48 h at 37°C, and growth quotients were determined. **(B)** Six-millimeter paper disks were placed on RPMI agar, or on RPMI agar at a final concentration of 100 µM or 1 mM FeCl_3_. Ten microliters of *A. fumigatus* 10AF conidia (10^7^/ml, prepared in RPMI) was placed on each disk, and 10 µl of *P. aeruginosa* bacteria (10^9^/ml, prepared in RPMI) was placed 15 mm distant from the disks carrying 10AF. Plates were incubated for 48 h at 37°C. **(C)** Six-millimeter paper disks were placed on RPMI agar or on iron-depleted RPMI agar (RPMI-IDA). Three disks were placed on each plate. Ten microliters of *A. fumigatus* 10AF conidia (10^7^/ml, prepared in RPMI) was placed on the middle disk, and 10 µl of *P. aeruginosa* bacteria (10^9^/ml, prepared in RPMI) was placed left and right in equal distance of 15 mm from the disks carrying 10AF. Plates were incubated for 24 h at 37°C. **(D)** Inhibition of fungal growth as shown in **(C)** was quantified by measuring 10AF growth angles (triple colony method). For each bar shown, two experiments were performed. Comparison for each group: PA14 (black bar) *vs*. PA14*pvdD-/pchE-* (gray bar), or as indicated by the ends of the bracket. Statistical analysis in **(A, D)**, t-test: two or three asterisks or pound signs = p ≤ 0.01 and p ≤ 0.001, respectively. Asterisks represent significant increases in antifungal activity; pound signs represent significant decreases.

So far, we observed that siderophore-deficient *P. aeruginosa* mutants had more antifungal activity on agar than the wild type, especially when both major siderophores pyoverdine and pyochelin were missing (PA14*pvdD-/pchE-*, PAO1*pvdA-/pchD-*). When pyoverdine was added to the PA14*pvdD/pchE* mutant, antifungal activity was reduced (**Figure 9A**), indicating that restoration of the ability to take up ferric iron reduced the antifungal activity. When 100 µM iron ferric iron was added to PA14 or PA14*pvdD*-/*pchE*- cocultures with *A. fumigatus*, antifungal activity on agar was reduced (**Figure 9B**). Concomitantly, on iron-depleted RPMI-agar (RPMI IDA), we found increased antifungal activity for the PA14 wild type, matching the activity of PA14*pvdD-/pchE-* (an example is shown in **Figure 9C**, and data are quantified in **Figure 9D**).

In addition to determining the effects of iron on antifungal activity on agar, we also determined the effects of calcium and magnesium. **Supplementary Figure 2** shows that in contrast to iron, neither calcium nor magnesium affected antifungal activity.

### Antifungal Activity of *P. aeruginosa *in Response to Ferrous Iron

**Figure 10:**
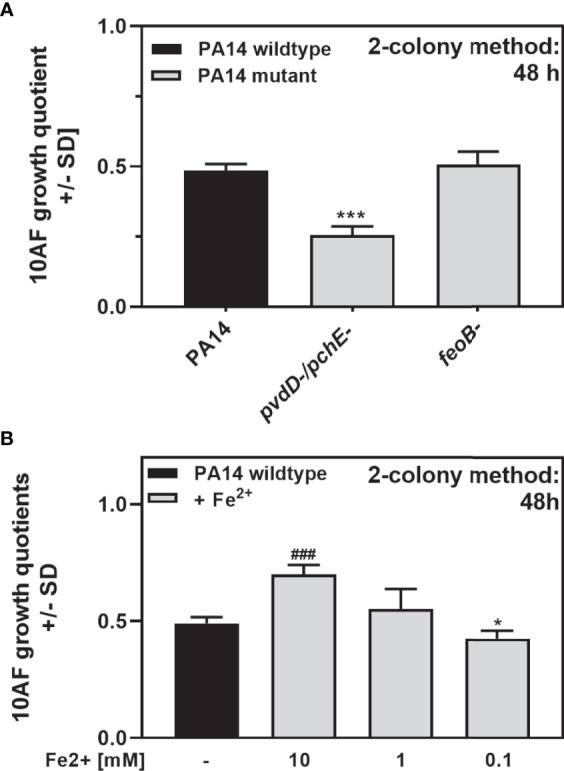
Antifungal activity of *P. aeruginosa* in response to ferrous iron. **(A)** Six-millimeter paper disks were placed on RPMI agar. Ten microliters of *A. fumigatus* 10AF conidia (10^7^/ml, prepared in RPMI) was placed on each disk, and 10 µl of PA14 wild-type, PA14*pvdD-/pchE-*, or PA14*feoB-* bacteria (10^9^/ml, prepared in RPMI) was placed 15 mm distant from the disks carrying 10AF. Plates were incubated for 48 h at 37°C, and growth quotients were determined. Comparison: PA14 (black bars) *vs*. all other bars in the same group. **(B)** Six-millimeter paper disks were placed on RPMI agar. Ten microliters of *A. fumigatus* 10AF conidia (10^7^/ml, prepared in RPMI) was placed on each disk, and 10 µl of ferrous iron was added to the filter disks at 10, 1, or 0.1 mM. Plates were incubated at 37°C for 48 h, and fungal growth areas were determined. Comparison: PA14 without iron addition (black bar) *vs*. all other bars. Statistical analysis in **(A, B)**, t-test: one or three asterisks or pound signs = p ≤ 0.05 and p ≤ 0.001, respectively. Asterisks represent significant increases in antifungal activity; pound signs represent significant decreases.

**Figure 9** shows that the availability of iron modulates antifungal activity on agar. Increased antifungal activity of PA14*pvdD-/pchE-* could be based on its inability to acquire ferric iron or triggered by its sole use of ferrous iron. A mutant defective in *feoB*, not able to use ferrous iron, showed no difference to the wild type in antifungal activity (**Figure 10A**), indicating that ferrous iron is not a factor in the increase of antifungal activity observed for PA14*pvdD-/pchE-*. Adding ferrous iron to PA14 decreased the antifungal activity at high concentrations (**Figure 10B**), indicating that iron, whether ferrous or ferric, counters the development of antifungal activity by *P. aeruginosa* on agar, presumably by relieving the stress of low iron. In summary, these experiments indicate that antifungal activity on agar is enhanced by a lack of iron.

## Discussion

In previous studies, we found that planktonic filtrates of *P. aeruginosa* have the ability to interfere with *A. fumigatus* metabolism *via* a number of its products, most noticeably *via* iron chelation by the *Pseudomonas* siderophore pyoverdine, withholding iron from the fungus (Sass et al., 2017; Sass et al., 2019; Nazik et al., 2020; Sass et al., 2021). In those studies, filtrates of a *P. aeruginosa* mutant defective in the production of both major siderophores, pyoverdine and pyochelin (PA14*pvdD-/pchE-*), showed the least antifungal activity (Sass et al., 2017). In the present study, we observed the opposite: the same mutant PA14*pvdD-/pchE-*, tested in the form of filtrates earlier, now was found most antifungal against *A. fumigatus* when both organisms were growing on agar in close proximity. Filtrates of this mutant, when placed next to a growing fungal colony on agar, did not interfere with growth, and neither did the wild-type filtrate (data not shown). The agar under and around a growing *P. aeruginosa* colony contained the substances responsible for antifungal activity on agar.

Therefore, the adaptation of growing on a solid surface and in an environment where bacteria are exposed directly to oxygen, and not dependent on diffusion through liquid, must have triggered *P. aeruginosa*, and even more so its double siderophore mutant, to produce molecules with antifungal activity, which are not pyoverdine, or pyochelin. Antifungal activity of PA14*pvdD-/pchE-* was stronger than that of the wild type, suggesting that the stress of iron shortage is an important factor for antifungal activity on agar.

Using *P. aeruginosa* mutants defective in quorum sensing (QS), we determined that QS plays an important role in antifungal activity on agar. Analysis of the mutants indicated that functions downstream of MvfR appear crucial for activity. We were able to overcome the loss of activity of a mutant of *pqsA*, a gene downstream of *mvfR*, by supplying PQS or HHQ, likely by *pqsA* involvement in activating transcription of the *pqsABCDE* operon. In contrast, we could not overcome the *mvfR* mutation with PQS or HHQ. Downstream molecules of MvfR are, e.g., phenazines, rhamnolipids, and proteases (Déziel et al., 2005; Diggle et al., 2006). The regulation of MvfR on the production of these molecules appears to be *via* the regulatory protein PqsE, which is encoded by the last gene of the *pqsABCDE* operon. PqsE participates in the regulation of production of molecules such as phenazines and rhamnolipids independently of the production of alkyl quinolones (Rampioni et al., 2016), *via* PqsE influencing RhlR activity. It is possible that the regulatory pathways in cultures on agar, particularly RPMI agar, could also differ from that in liquid cultures, where the current understanding of the pathways was delineated. Thus, our understanding of our results is that antifungal signaling on a surface is a combination of PqsE effects and alkyl quinolone activities, *via* the alkyl quinolones intensifying the effects of *mvfR* downstream products or activating transcription of the operon. Using specific downstream mutants as well as pure molecules, we determined that rhamnolipids and elastase are major molecules in antifungal activity on agar.

High concentrations of ferric iron or the addition of pyoverdine lessened the antifungal activity of PA14*pvdD/pchE*. The presumed mechanism is *via* relief of the low-iron stresses. On iron-depleted agar wild-type *P. aeruginosa* gained antifungal activity.

We could demonstrate that PA14*pvdD*-/*pchE*-, being more antifungal than wild-type *P. aeruginosa* on agar, produced significantly higher amounts of rhamnolipids and elastase than the wild type. Ferric iron reduces rhamnolipid production by *P. aeruginosa* (Déziel et al., 2005; Yu et al., 2016), whereas iron limitation enhances rhamnolipid production. Similarly, high iron concentrations interfered with elastase production by some *P. aeruginosa* strains (Bjorn et al., 1979), whereas iron-limiting conditions increased the production of elastase (Kim et al., 2003). Rhamnolipids and elastase are *P. aeruginosa* molecules that could, by activity on substrates in its environment, or by actions on its competitors, help *P. aeruginosa* in the acquisition of iron.

Both elastase and rhamnolipids in our study were found to be antifungal, and complementation of the respective mutants increased antifungal activity to levels seen for the wild type. It has to be taken into account that we used small amounts of rhamnolipids and elastase to interfere with fungal growth in our assays and that we applied pure molecules only once before incubating cultures for 48 h at 37°C. Elastase, especially, might degrade substantially during this incubation period. In contrast to single applications, continuous production from a *P. aeruginosa* colony would also be expected to have greater effects.

We and others (Haba et al., 2003; Benincasa et al., 2004; Van Gennip et al., 2009; Sha et al., 2012; Briard et al., 2017) observed antifungal activity of rhamnolipids, which seems to be based either on surfactant activity of rhamnolipids or on cell wall thickening and inhibition of fungal growth, especially by di-rhamnolipids (Briard et al., 2017). We observed that antifungal activities on agar predominantly were driven by mono-rhamnolipids. In the mammalian host, rhamnolipids contribute to fungal biofilms and lysis of immune cells that might augment antifungal effects (Alhede et al., 2014). Elastase had not yet been described to have activity against *A. fumigatus*, although it is known that *A. fumigatus* enhances the production of elastase by *P. aeruginosa* in cocultures (Smith et al., 2015). In addition, we observed that elastase and rhamnolipids, when combined with growing bacterial cultures in combination, further increased the antifungal activity of the PA14 wild type. It has to be noted that each factor alone, although complementing the antifungal activity of its mutant, did not increase antifungal activity of the wild type.

The finding that low iron conditions increased the antifungal activity of *P. aeruginosa* on agar are not restricted to PA14 but also apply to PAO1, where a double siderophore mutant was also more antifungal than the wild type, and QS mutants were less antifungal than the wild type as well. Also, in the PAO1 background the double siderophore mutant produced more rhamnolipids than its wild type, similar to our observations shown for PA14 and its double siderophore mutant. We also determined that antifungal effects did not depend on the *A. fumigatus* strain tested, as similar antifungal effects were seen when using 10AF or Af293.

Exotoxin A production has shown iron dependence (Bjorn et al., 1978). As **Supplementary Table 1** and **Figure 4B** show, mutation to ablate exotoxin A production (*via* mutation of pvdS) in the PAO1 background does not remove antifungal activity. This indicates that exotoxin A is not a factor in the iron-dependent antifungal activity on agar.

In a previous study (Nazik et al., 2020), we showed that volatiles produced by *P. aeruginosa* colonies could inhibit *A. fumigatus* colonies *via* the production of small organic molecules. This method of inhibition appears to minimally contribute, if at all, to the inhibition emanating from *P. aeruginosa* colonies in the circumstances (quantities of microbes, kinetics of inhibition, use of agar unfavorable for volatile production) of the present studies. We observed here directionality of the inhibition (greatest near the *P. aeruginosa* colony), whereas volatiles reduced *A. fumigatus* growth uniformly and circumferentially. The profile of inhibition by *P. aeruginosa* mutants *via* volatiles does not generally match the profile of inhibition shown on agar (e.g., **Figure 3**), and particularly the double siderophore mutant was equal to the wild type in inhibition *via* volatiles, not greater, as in the present studies. In both studies, however, the most impaired QS mutants lacked inhibitory activity. Finally, iron was not a factor in the amount of volatiles produced.

In summary, iron is a crucial factor that affects the antifungal activity of *P. aeruginosa*. A lack of iron causes *P. aeruginosa* to produce more rhamnolipids and elastase, which at a close proximity to the bacterial culture interfere with fungal growth. A lack of iron also induces the production of pyoverdine and other siderophores which complex the remaining iron in the medium, deprive the fungus of this crucial factor, and provide iron to *P. aeruginosa*. Whereas *P. aeruginosa* also shows antifungal activity under high iron conditions in liquid medium mainly *via* production of phenazines (Sass et al., 2021), antifungal activity of *P. aeruginosa* on agar in close proximity to the fungus is diminished by iron. Such short-range antifungal activity of *P. aeruginosa* is enhanced when bacterial siderophores are missing, i.e., when low-iron stress is most evident. These observations add to the literature emphasizing the versatility of *P. aeruginosa* in intermicrobial competition.

## Data Availability Statement

The raw data supporting the conclusions of this article will be made available by the authors, without undue reservation.

## Author Contributions

GS: execution, supervision, data curation, analysis, writing first draft, revision HN: execution, analysis, revision PC: execution, analysis, revision PS: execution, revision M-CG: execution, analysis, revision ED: supervision, data curation, analysis, revision DS: supervision, data curation, analysis, financial support, revision. All authors contributed to the article and approved the submitted version.

## Funding

These studies were partially supported by the Foundation for Research in Infectious Diseases (FRID), CIMR no. 8201. The funder had no role in study design, data collection and interpretation, or the decision to submit the work for publication.

## Conflict of Interest

The authors declare that the research was conducted in the absence of any commercial or financial relationships that could be construed as a potential conflict of interest.

## Publisher’s Note

All claims expressed in this article are solely those of the authors and do not necessarily represent those of their affiliated organizations, or those of the publisher, the editors and the reviewers. Any product that may be evaluated in this article, or claim that may be made by its manufacturer, is not guaranteed or endorsed by the publisher.

## Supplementary Material

Supplementary Table 1
*P. aeruginosa* mutants used in this study.
