# Supplementary material for: Altered Pseudomonas Strategies to Inhibit Surface Aspergillus Colonies
Source: Front Cell Infect Microbiol. 2021 Oct 22;11:734296. doi: 10.3389/fcimb.2021.734296 (PMC8570168; doi:10.3389/fcimb.2021.734296)
Supplement: Supplementary Table 1 — P. aeruginosa mutants used in this study. [file Table_1.docx]

**Supplemental Table 1:** ***P. aeruginosa* mutants used in this study**

| **Mutant in:** | **result of mutation** | **Ref.** |
| --- | --- | --- |
| **PA14** |  |  |
| *pvdD/*  *pchE* | Pyoverdin-pyochelin double siderophore mutant | Sass et al., 2017 |
| *pvdD* | Loss of pyoverdin (siderophore) | Liberati et al., 2006 |
| *pchE* | Loss of pyochelin | Liberati et al., 2006 |
| *feoB* | Loss of ability to use Fe^2+^ | Liberati et al., 2006 |
| *pqsH* | Loss of 2-heptyl-3-hydroxy-4(1H)-quinolone synthase; loss of PQS biosynthesis, thus decreased activity of MvfR; MvfR not as negatively affected than for pqsA-, since pqsH- still produces HHQ and other HAQs that can act as ligands of PQS | Xiao et al., 2006 |
| *pqsA/*  Δ*pqsH NOT polar* | Same predicted outcome as pqsA-. In both, the *pqsA-* mutation is nonpolar on downstream genes in the operon, thus not preventing their transcription. | Xiao et al., 2006 |
| *pqsA/*  *pqsH polar* | pqsA::TnPhoA, pqsH::Gm, the mutation in pqsA is polar (thus theoretically preventing transcription of downstream genes in the operon), Kan and Gm resistant | @ |
| *pqsE* | Gene mediates the regulatory activity of the MvfR system (see PA14Δ*mvfR*); extracellular quinolones similar to wild type, defective in pyocyanin | Déziel et al., 2004 |
| *pqsA* | Gene for anthranilate-CoA ligase lost; loss of extracellular quinolones, HAQ biosynthesis, including HHQ, PQS, DHQ; thus absent or decreased activity of MvfR | Déziel et al., 2004 |
| *pqsL* | Overproduction of the alkyl quinolone PQS. | Lépine et al., 2004 |
| *mvfR* | Under-production of various QS-regulated factors related to loss of HAQs, phenazines, proteases, HCN, lectins and others | Cao et al., 2001 |
| *lasR/*  *rhlR* | Double mutant defective for most QS-regulated metabolites, including phenazines, rhamnolipids, AHL, HAQs, proteases, HCN, chitinase, elastase, less pyoverdine | Dekimpe et al., 2009 |
| *lasR* | Lacks several QS-regulated factors, including proteases, oxo-C_12_-HSL; delayed activation of RhlR QS pathway, less pyoverdine | Déziel et al., 2004 |
| *lasI* | No C_12_-HSL production | Liberati et al., 2006 |
| *lasA* | Loss of protease | Liberati et al., 2006 |
| *lasB* | Loss of elastase | Liberati et al., 2006 |
| *rhlA* | Loss of rhamnolipids | Ochsner et al., 1994 |
| *rhlB* | Loss of rhamnolipids | Liberati et al., 2006 |
| *rhlR* | Loss of rhamnolipids, phenazines, HCN, lectins, C_4_-HSL | [Déziel](https://pubmed.ncbi.nlm.nih.gov/?sort=pubdate&term=D%C3%A9ziel+E&cauthor_id=15686549) et al., 2005 |
| *phzC1*/  *phzC2* | Double phenazine mutant; minimal residual phenazine production | @ |
| *phzM* | Defective in phenazine-specific methyltransferase, no pyocyanin | * |
| *phzH* | Defective in phenazine pathway, no PCN | Liberati et al., 2006 |
| *phzS* | Defective in phenazine-specific flavin-containing monooxygenase, no 1HP and no pyocyanin | Liberati et al., 2006 |
| *HSI/II* | Double mutant defective in 2 of 3 type VI secretion systems | Lesic et al., 2009 |
| *HSI/III* | Double mutant defective in 2 of 3 type VI secretion systems | Lesic et al., 2009 |
| *HSII/III* | Double mutant defective in 2 of 3 type VI secretion systems | Lesic et al., 2009 |
| *plcH* | Heat-labile hemolytic phospholipase C (plcH) mutant. | Liberati et al., 2006 |
| *plcN* | Non-hemolytic phospholipase C (plcN) mutant. | Liberati et al., 2006 |
| *rsmA* | Global post-transcriptional regulator mutant | Liberati et al., 2006 |
| *rsmYZ* | Genes for coding small regulatory RNAs lost, decreased production of C_4_-HSL, phenazines, chitinase | Sass et al., 2017 |
| *pvcA* | Loss of paerucumarin and pseudoverdin | Liberati et al., 2006 |
| *exoU* | Loss of exotoxin U | Liberati et al., 2006 |
| *chiC* | Chitinase C mutant | Liberati et al., 2006 |
| *lecA* | Lectin A mutant | Liberati et al., 2006 |
| *hcnA* | HCN mutant, loss of hydrogen cyanide | Liberati et al., 2006 |
| *pscC* | Defective in all 3 secretion systems | Liberati et al., 2006 |
| **PAO1** |  |  |
| *pvdA* | type-I pyoverdine defective | Minandri et al., 2016 |
| *pvdA/*  *fpvR* | pyoverdine-defective, but still able to produce PvdS-dependent virulence factors (Exotoxin A, PrpL protease) | Minandri et al., 2016 |
| *pvdS* | pyoverdine-defective and unable to produce PvdS-dependent virulence factors (Exotoxin A, PrpL protease) | Minandri et al., 2016 |
| *pvdA/*  *pchD* | pyochelin and pyoverdine defective | ** |
| *pchD* | pyochelin defective | ** |
| *lasR/*  *rhlR* | Double mutant defective for most QS-regulated metabolites, including phenazines, rhamnolipids, AHL, HAQs, proteases, HCN, chitinase, elastase, less pyoverdine | ** |
| *pqsA* | Gene for anthranilate-CoA ligase lost; loss of extracellular quinolones, HAQ biosynthesis, including HHQ, PQS, DHQ; thus absent or decreased activity of MvfR | ** |

@: Unpublished lab strain (available from E. Déziel)

* kindly provided by P. Cornelis; ** kindly provided by P. R. Secor

Abbreviations: QS: quorum-sensing; HAQ: 4-hydroxy-2-alkylquinolones; DHQ: 2,4-dihydroxyquinoline, MvfR: Multiple Virulence Factor Regulator, HCN: hydrogen cyanide; 1HP: 1-hydroxyphenazine, AHL: acylhomoserine lactones; HSL: homo serine lactone; HHQ: 4-hydroxy-2-heptylquinoline; PQS: 3,4-dihydroxy-2-heptylquinoline (Lépine et al., 2004), Gm: gentamicin, Kan: kanamycin, CoA: coenzyme A.

Cao, H., Krishnan, G., Goumnerov, B., Tsongalis, J., Tompkins, R., Rahme, L.G. (2001). A Quorum Sensing-Associated Virulence Gene of *Pseudomonas Aeruginosa* Encodes a LysR-like Transcription Regulator with a Unique Self-Regulatory Mechanism. Proc. Natl. Acad. Sci. U S A 98, 14613-14618.

Dekimpe, V. and Déziel, E. (2009). Revisiting the Quorum-Sensing Hierarchy in *Pseudomonas Aeruginosa*: The Transcriptional Regulator RhlR Regulates LasR-Specific Factors. Microbiology 155, 712-723.

Déziel, E., Lépine, F., Milot, S., He, J., Mindrinos, M.N., Tompkins, R.G., et al. (2004). [Analysis of *Pseudomonas Aeruginosa* 4-hydroxy-2-alkylquinolines (HAQs) Reveals a Role for 4-hydroxy-2-heptylquinoline in Cell-to-Cell Communication.](https://www.ncbi.nlm.nih.gov/pubmed/14739337) Proc. Natl. Acad. Sci. U S A 101, 1339-1344.

[Déziel](https://pubmed.ncbi.nlm.nih.gov/?sort=pubdate&term=D%C3%A9ziel+E&cauthor_id=15686549), E. , [Gopalan](https://pubmed.ncbi.nlm.nih.gov/?sort=pubdate&term=Gopalan+S&cauthor_id=15686549), S.,  [Tampakaki](https://pubmed.ncbi.nlm.nih.gov/?sort=pubdate&term=Tampakaki+AP&cauthor_id=15686549), A.P., [Lépine](https://pubmed.ncbi.nlm.nih.gov/?sort=pubdate&term=L%C3%A9pine+F&cauthor_id=15686549), F.,  [Padfield](https://pubmed.ncbi.nlm.nih.gov/?sort=pubdate&term=Padfield+KE&cauthor_id=15686549), K.E., [Saucier](https://pubmed.ncbi.nlm.nih.gov/?sort=pubdate&term=Saucier+M&cauthor_id=15686549), et al. (2005). The Contribution of MvfR to *Pseudomonas Aeruginosa* Pathogenesis and Quorum Sensing Circuitry Regulation: Multiple Quorum Sensing-Regulated Genes are Modulated without Affecting lasRI, rhlRI or the Production of N-acyl-L-homoserine Lactones. Mol. Microbiol. 55, 998-1014. doi: 10.1111/j.1365-2958.2004.04448.x.

Lépine, F., Déziel, E., Milot, S., Rahme, L.G. (2003). [A Stable Isotope Dilution Assay for the Quantification of the *Pseudomonas* Quinolone Signal in *Pseudomonas Aeruginosa* Cultures.](https://www.ncbi.nlm.nih.gov/pubmed/12829259) Biochim. Biophys. Acta*.*1622, 36-41.

Lépine, F., Milot, S., Déziel, E., He, J., Rahme, L.G. (2004). [Electrospray/Mass Spectrometric Identification and Analysis of 4-hydroxy-2-alkylquinolines (HAQs) Produced by *Pseudomonas Aeruginosa.*](https://pubmed.ncbi.nlm.nih.gov/15144975/) J. Am. Soc. Mass Spectrom. 15:862-869. doi: 10.1016/j.jasms.2004.02.012.

[Lesic, B](https://www.ncbi.nlm.nih.gov/pubmed/?term=Lesic%20B%5BAuthor%5D&cauthor=true&cauthor_uid=19497948)., [Starkey, M](https://www.ncbi.nlm.nih.gov/pubmed/?term=Starkey%20M%5BAuthor%5D&cauthor=true&cauthor_uid=19497948)., [He, J](https://www.ncbi.nlm.nih.gov/pubmed/?term=He%20J%5BAuthor%5D&cauthor=true&cauthor_uid=19497948)., [Hazan, R](https://www.ncbi.nlm.nih.gov/pubmed/?term=Hazan%20R%5BAuthor%5D&cauthor=true&cauthor_uid=19497948)., [Rahme, L.G](https://www.ncbi.nlm.nih.gov/pubmed/?term=Rahme%20LG%5BAuthor%5D&cauthor=true&cauthor_uid=19497948). (2009). Quorum Sensing Differentially Regulates *Pseudomonas Aeruginosa* Type VI Secretion Locus I and Homologous Loci II and III, which are Required for Pathogenesis. [Microbiology](https://www.ncbi.nlm.nih.gov/pubmed/?term=Lesic+Starkey+He+Hazan+Rahme) 155, 2845-2855.

Liberati, N.T., Urbach, J.M., Miyata, S., Lee, D.G., Drenkard, E., Wu, G., et al. (2006). [An Ordered, Nonredundant Library of *Pseudomonas Aeruginosa* Strain PA14 Transposon Insertion Mutants.](https://www.ncbi.nlm.nih.gov/pubmed/16477005) Proc. Natl. Acad. Sci. U S A 103, 2833-2838.

Minandri, F., Imperi, F., Frangipani, E., Bonchi, C., Visaggio, D., Facchini, M., et al. (2016). Role of Iron Uptake Systems in *Pseudomonas Aeruginosa* Virulence and Airway Infection. Infect. Immun. 84, 2324-2335.

Ochsner, U.A., Fiechter, A., Reiser, J. (1994). [Isolation, Characterization, and Expression in *Escherichia Coli* of the *Pseudomonas Aeruginosa* rhlAB Genes Encoding a Rhamnosyltransferase Involved in Rhamnolipid Biosurfactant Synthesis.](https://www.ncbi.nlm.nih.gov/pubmed/8051059) J. Biol. Chem. 269, 19787-19795.

Sass, G., Nazik, H., Penner, J., Shah, H., Ansari, S.R., Clemons, K.V., et al. (2017). [Studies of *Pseudomonas Aeruginosa* Mutants Indicate Pyoverdine as the Central Factor in Inhibition of *Aspergillus Fumigatus* Biofilm.](https://www.ncbi.nlm.nih.gov/pubmed/29038255) J. Bacteriol. 200, e00345-17. doi: 10.1128/JB.00345-17.

Xiao, G., Déziel, E., He, J., Lépine, F., Lesic, B., Castonguay, M.H., et al. (2006). [*MvfR, a Key Pseudomonas Aeruginosa* Pathogenicity LTTR-class Regulatory Protein, has Dual Ligands.](https://www.ncbi.nlm.nih.gov/pubmed/17083468) Mol. Microbiol. 62, 1689-1699.
