# Supplementary material for: Altered Pseudomonas Strategies to Inhibit Surface Aspergillus Colonies
Source: Front Cell Infect Microbiol. 2021 Oct 22;11:734296. doi: 10.3389/fcimb.2021.734296 (PMC8570168; doi:10.3389/fcimb.2021.734296)
Supplement: Supplementary file 2 [file DataSheet_1.pdf]

Supplemental Figure 1

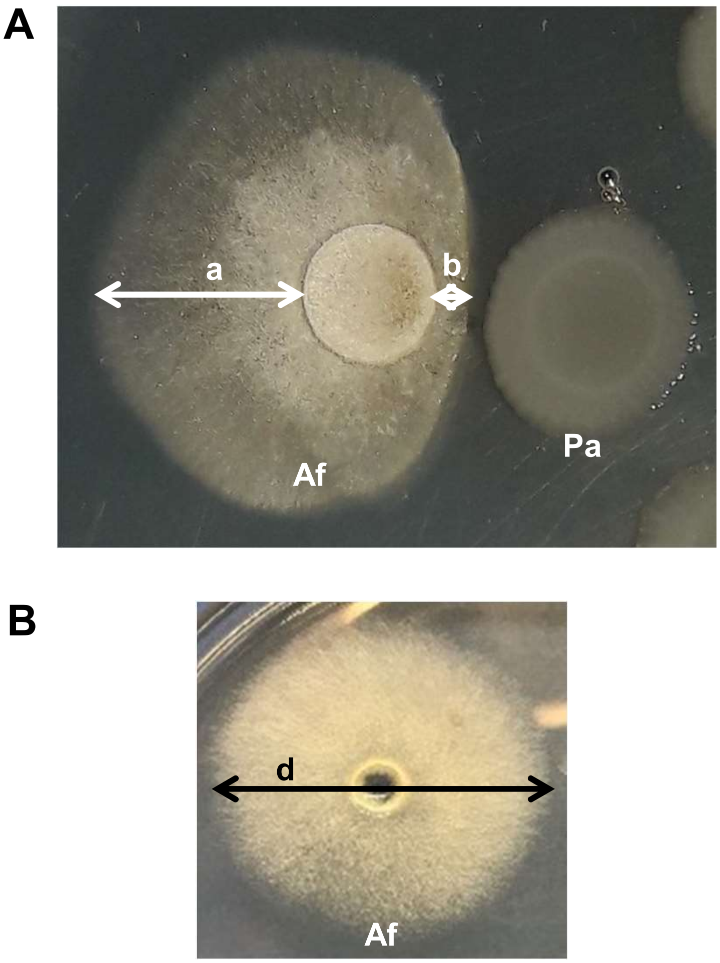

Supplemental Figure 1: Visualization of A. fumigatus growth measurement methods

A: 2-colony method: Sterile paper discs were placed on RPMI agar or TSA plates, and inoculated with 10 µl of Af suspension, [10^7 conidia/ml RPMI]. Ten µl of *P. aeruginosa* suspension, [10^9 cell/ml RPMI] were inoculated at 15 mm distance from the center of the Af inoculation point directly onto the agar. Plates were incubated at 37°C for 48h, unless indicated otherwise. To quantify effects of nearby *P. aeruginosa* colony growth on *A. fumigatus* colony growth a quotient was calculated by dividing the distance from the edge of the filter disk to the end of fungal growth in the direction of the bacterial colony (b) by the distance from the end of the filter disk to the end of fungal growth in the opposite direction (a). For undisturbed fungal growth (growth in the presence of 10 µL of RPMI) this quotient = 1, for inhibition of fungal growth the quotient would be < 1, for stimulation of fungal growth the quotient would be > 1.

B: Determination of the *A. fumigatus* growth zone: d: diameter. The growth zone was determined following the formula: $r^2\pi$. The area of inoculation (wells or disks, 28 mm^2) was subtracted from the calculated fungal growth areas.

Supplemental Figure 2

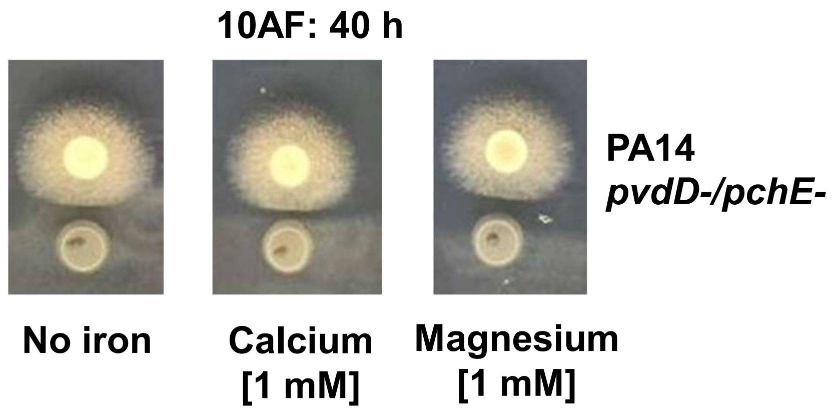

Supplemental Figure 2: Effects of calcium and magnesium on anti-fungal activity of PA14*pvdD/pchE* on agar

Six-mm paper disks were placed on RPMI agar, or on RPMI agar containing 1 mM $CaCl_2$, or 1 mM $MgCl_2$. Ten µl of *A. fumigatus* 10AF conidia (10^7/ml, prepared in RPMI) was placed on each disk, and 10 µl of *P. aeruginosa* bacteria (10^9/ml, prepared in RPMI) was placed 15 mm distant from the disks carrying 10AF. Plates were incubated for 40 hr at 37°C.
